# Deletion of the gene for the African swine fever virus BCL-2 family member A179L increases virus uptake and apoptosis but decreases virus spread in macrophages and reduces virulence in pigs

**DOI:** 10.1128/jvi.01106-23

**Published:** 2023-10-05

**Authors:** Ana Luisa Reis, Anusyah Rathakrishnan, Leah V. Goulding, Claire Barber, Lynnette C. Goatley, Linda K. Dixon

**Affiliations:** 1 The Pirbright Institute, Woking, Surrey, United Kingdom; Lerner Research Institute, Cleveland, Ohio, USA

**Keywords:** African swine fever virus, apoptosis, A179L, BCL-2, macrophages, virulence, Annexin V

## Abstract

The African swine fever virus (ASFV) encodes inhibitors of innate immune responses, including type I interferon and apoptosis. The BCL-2 family A179L gene was deleted from the virulent genotype I isolate Benin 97/1. In infected macrophages, this resulted in increased caspase 3 and 7 activities, Annexin V binding to surface phosphatidylserine, and DNA fragmentation, measured by terminal deoxynucleotidyl transferase nick-end labeling, compared to Benin 97/1 expressing A179L or mock-infected macrophages. These results confirmed that apoptosis was induced earlier in macrophages infected with viruses from which A179L was deleted. Increased cell entry of the A179L gene-deleted virus was indicated since the number of infected cells at early times, as measured by fluorescent protein expression, was much higher than infection with the wild-type virus. This increase in entry was reduced following blocking of the virus by binding to Annexin V, suggesting that entry was mediated by phosphatidylserine receptors. Yields of infectious viruses were similar over a single cycle but significantly lower for the A179L gene-deleted virus over a multistep growth cycle. Pigs immunized with the BeninΔA179L virus showed no clinical signs and a weak cellular response to ASFV. The immunized pigs were not protected against challenge with the virulent parental virus Benin 97/1 although viremia was lower at 3 d post-challenge than the control nonimmune pigs. Thus, reduced levels of virus replication in pigs limited the induction of a protective immune response. The results show an important role for the A179L protein in virus replication in macrophages and virulence in pigs.

**IMPORTANCE:**

African swine fever virus (ASFV) causes a lethal disease of pigs with high economic impact in affected countries in Africa, Europe, and Asia. The virus encodes proteins that inhibit host antiviral defenses, including the type I interferon response. Host cells also activate cell death through a process called apoptosis to limit virus replication. We showed that the ASFV A179L protein, a BCL-2 family apoptosis inhibitor, is important in reducing apoptosis in infected cells since deletion of this gene increased cell death and reduced virus replication in cells infected with the A179L gene-deleted virus. Pigs immunized with the BeninΔA179L virus showed no clinical signs and a weak immune response but were not protected from infection with the deadly parental virus. The results show an important role for the A179L protein in virus replication in macrophages and virulence in pigs and suggest manipulation of apoptosis as a possible route to control infection.

## INTRODUCTION

African swine fever (ASF) is a highly contagious disease of domestic pigs and wild boars caused by a double-stranded DNA virus, African swine fever virus (ASFV) (1, 2). ASFV is the only member of the Asfarviridae family and replicates in the cytoplasm of cells of the myeloid lineage, primarily macrophages or monocytes, at an intermediate or late stage of differentiation (3). In East Africa, ASFV is maintained in a sylvatic cycle involving warthogs (*Phacochoerus africanus*) and soft ticks of the genus *Ornithodoros* (4). The virus can persist in warthogs and bushpigs with few clinical signs of disease, but case fatality rates in domestic pigs or wild boars infected with virulent isolates of ASFV can approach 100% (5 – 8). In 2007, ASFV was introduced to the Republic of Georgia (9) and spread to Russia, Eastern Europe, and European Union member countries. Following its introduction to China in 2018, the disease spread extensively there and in other countries in Southeast Asia, causing high economic losses (10 – 12). Vaccines are not widely available, thereby limiting the control of ASFV outbreaks. Ongoing efforts center on the targeted deletion of nonessential virus genes to construct modified live-attenuated vaccines (13 – 17).

The ASFV genome varies between 170 and 193 kbp and encodes up to 170 proteins. Many of the virus genes are not essential for replication but play important roles in modulating the host’s antiviral defenses. Among these are inhibitors of the type I interferon response and inhibitors of apoptosis (1, 18). Apoptosis of infected host cells is an effective protective mechanism to disrupt virus replication and limit the spread of progeny viruses (19). ASFV encodes several regulators of host apoptotic pathways, including an inhibitor of apoptosis (IAP) protein homolog (A224L) that inhibits caspase 3 activity, an inhibitor of the pro-apoptotic stress-induced CHOP protein-dependent pathway (DP71L), a viral C-type lectin that inhibits p53 activity (EP153R), and a viral BCL-2 protein family member (A179L) (20 – 23).

The BCL-2 family proteins regulate the host cell’s commitment to apoptosis through interplay between each other. Ultimately, mitochondrial outer membrane permeabilization is regulated via pores formed by pro-apoptotic BCL-2 family proteins and the subsequent release of apoptotic mediators into the cytosol. The BCL-2 family members are broadly divided into the antiapoptotic or “pro-survival” members and the pro-apoptotic family members. The antiapoptotic members include BCL-2, BCL-x_L_, MCL-1, BCL-W, and BFL-1/A1 (24, 25). The pro-apoptotic members include pore-forming proteins (BAX, BAK, and BOK) and the BH3-only proteins (such as BAD, BID, and BIK) (26, 27). The BH3-only proteins function either by binding and “activating” the pore-forming proteins or through binding to antiapoptotic proteins and “freeing” sequestered pro-apoptotic regulators (27, 28). Antiapoptotic BCL-2 proteins sequester the pro-apoptotic family members to prevent permeabilization of the mitochondrial membrane (24, 25). Several viruses code for homologs of the antiapoptotic BCL-2 family members to regulate apoptosis (29 – 31).

The ASFV A179L protein has antiapoptotic activity when expressed exogenously in cells and is proposed to promote cell survival to facilitate ASFV replication (21, 32), although until now this activity has not been demonstrated in ASFV-infected cells. A179L contains three BCL-2 homology domains (BH4, BH1, and BH2) and localizes to the mitochondria or endoplasmic reticulum (33, 34). The protein associates with pro-apoptotic BCL-2 proteins (such as Bid, Bim, and Bad) with varying affinities, in addition to the autophagy-associated protein Beclin-1 (35 – 37). A179L, like the antiapoptotic BCL-2 family members such as BCL-2 and BCL-x_L_, may inhibit apoptosis through association with the BH3 motifs of the pro-apoptotic regulators, thereby interfering with their activity and inhibiting cell death to extend virus replication (36).

To further understand the role of the A179L protein during ASFV infection, we inactivated the A179L gene on the genome of the virulent ASFV isolate Benin 97/1. This resulted in a 10-fold reduction in Benin 97/1 replication in porcine primary macrophages over a multistep growth curve. Comparison of infections between BeninΔA179L and the parental virus showed increased activation of caspases 3 and 7 and of DNA fragmentation measured by terminal deoxynucleotidyl transferase nick-end labeling (TUNEL). In addition, increased binding of Annexin V to the cell surface was observed in the early stages post-infection of viruses from which the A179L gene was deleted when compared to viruses with intact A179L. These results support the conclusion that A179L suppresses caspase activation and apoptosis in infected macrophages. Live cell imaging of macrophages infected with wild-type or A179L gene-deleted viruses expressing fluorescent proteins showed a much greater infection rate in the A179L deletion mutant. This may result from the incorporation of phosphatidylserine into the external envelope and uptake mediated by phosphatidylserine receptors. We also showed that the deletion of the A179L gene attenuated the Benin 97/1 virus since no clinical signs or viremia were observed following infection. However, the pigs were not protected against challenge with the parental virulent virus, suggesting that insufficient virus replication had occurred to induce a protective response. The results show that A179L plays an important role in ASFV replication in macrophages and virulence in pigs.

## RESULTS

### Generation of A179L recombinant viruses

**FIG 1:**
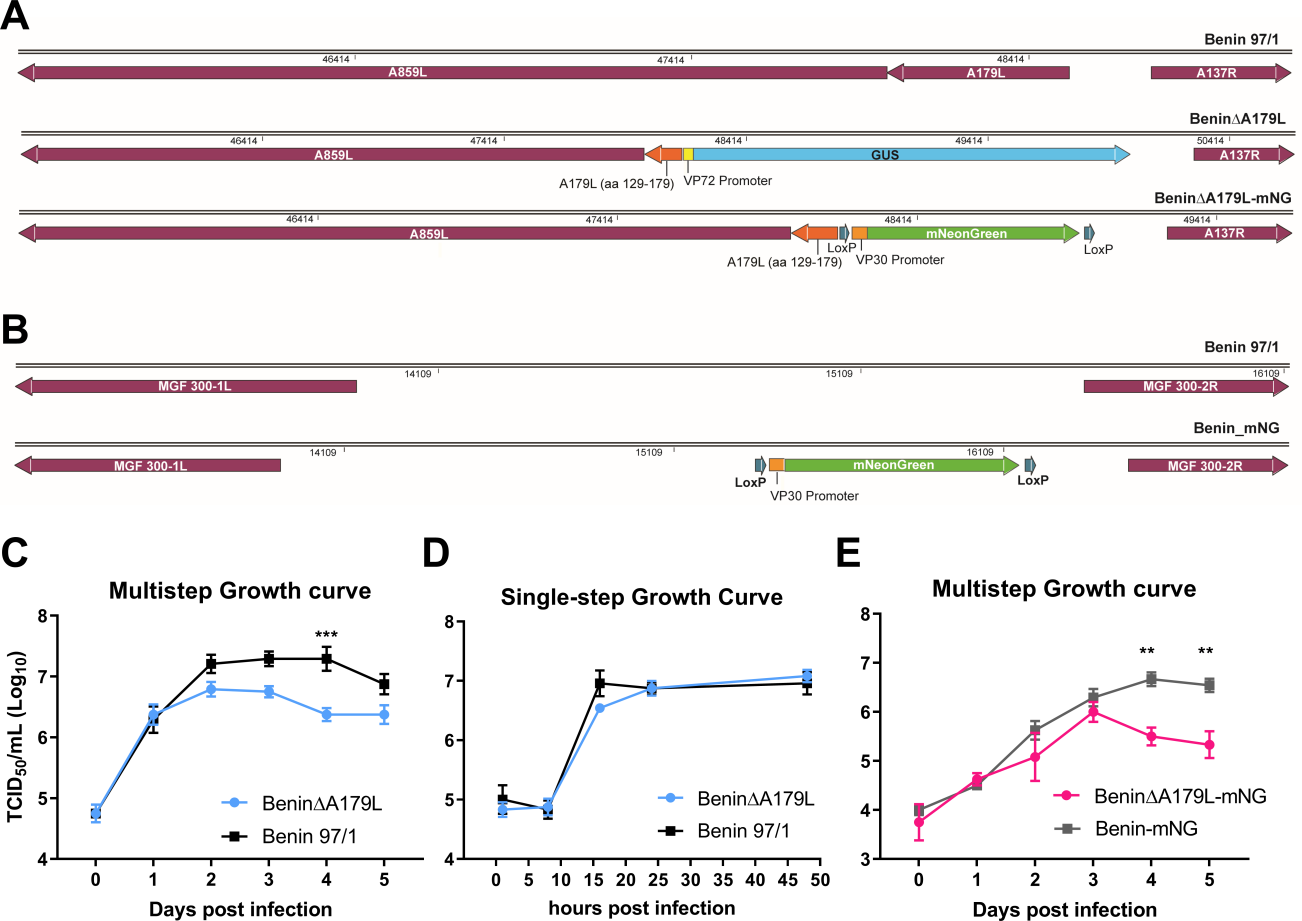
Deletion of the A179L gene from the ASFV Benin 97/1 strain. (**A**) A schematic diagram depicting the deletion of A179L from the Benin 97/1 ASFV isolate is shown. In the top panel, the position of the A179L gene and flanking genes A859L and A137R are shown. In the middle panel, a diagram of the genome with the A179L gene deleted and the β-GUS reporter gene under control of the ASFV p72 promoter inserted in the genome is shown. Note that a fragment of the A179L gene (coding for residues 129 to 179) is retained in the genome to avoid disrupting the promoter for the downstream A859L gene. In the bottom panel, the same A179L gene deletion is shown, and instead of the β-GUS reporter gene, a fluorescent reporter gene, mNeonGreen, under control of the ASFV p30 promoter, was inserted. (**B**) The mNeonGreen gene under control of the ASFV p30 promoter was inserted in the noncoding region of Benin 97/1 at position 15,358 between genes MGF300-1L and MGF300-2R to produce a fluorescent-tagged Benin 97/1. Purified PBMs from two different pigs were infected with viruses at an MOI of 0.01 in triplicates over 5 d (**C, E**) or at an MOI of 1.0 for 48 h (**D**). Viruses were harvested from both cells and supernatants at different time points indicated (x-axis) and titrated on Vero or PBMs in quadruplicates (y-axis). Day 0 shows virus in the inoculum (**C-E**). A two-way analysis of variance with Šídák’s multiple comparisons test was performed to evaluate the differences between the recombinant viruses and the wild-type viruses. Significant differences are represented by an asterisk, where ** is P < 0.01 and *** is *P* < 0.001.

To delete the A179L gene from Benin 97/1, a transfer vector pcDNA3_A179L-LF_VP72-GUS_A179L-RF was constructed to include the left and right flanking regions of A179L from the ASFV genome. The β-glucuronidase (GUS) reporter gene, under control of the VP72 (B646L) gene promoter, was inserted between the flanking regions, replacing sequences from the A179L gene (Fig. 1A). A179L is read toward the left genome end and is separated by a single base between the stop codon of A179L and the start codon of A859L. We therefore incorporated 157 bp of the 3′ end of the A179L gene into the left flank of the transfer vector so that the promoter of A859L would not be interrupted. The A859L gene is expressed late during infection, and its promoter region may be within the 3′ end of the A179L coding region, which is expressed early (38). A859L codes for a protein belonging to helicase superfamily 2. Deletion of this gene from a virulent ASFV isolate, Georgia/2007, did not reduce virus replication in macrophages or virulence in pigs (39), so any changes in its expression would not be expected to affect virus growth. The 157-bp region of A179L included in the transfer vector contains the sequence that encodes residues at the C-terminus of A179L from 129 to 179 amino acids. However, this is unlikely to be expressed as there is no in-frame upstream translation start codon. The 157-bp fragment contains sequences coding for most of the C-terminal BH2 and lacks just the two N-terminal residues from this domain. However, the upstream BH4 and BH1 domains are not present in the 157-bp fragment remaining from the A179L gene. Structural analysis of A179L bound to BH3 domains from BID and BAX showed that residues encompassing all three BH domains of A179L between residues 2 and 141 are involved in binding these pro-apoptotic proteins. Specific residues, including R86, G76, and Y86, are important for binding to BH3 domain proteins. Therefore, in the unlikely event that this fragment remaining at the C-terminus of A179L was expressed, it is unlikely to be functional. The transfer vector was transfected into porcine alveolar macrophages (PAMs) infected with Benin 97/1 virus, and the recombinant virus BeninΔA179L was identified by expression of the reporter gene in infected cells and purified by limiting dilution over 10 passages. Two additional recombinant viruses were constructed, each expressing an mNeonGreen fluorescent protein, in order to follow infections of macrophages by live cell imaging. In one of these, the A179L gene was deleted from the Benin 97/1 virus using the same flanking regions as for the GUS-expressing mutant virus but using mNeonGreen under control of the ASFV p30 (CP204L) gene promoter as a reporter to isolate recombinant viruses by single cell sorting (40). This recombinant virus is referred to as BeninΔA179L-mNG (Fig. 1A). To provide a control virus, the mNeonGreen reporter was inserted at position 15,358 in a noncoding area of the Benin 97/1 genome between MGF300-1L and MGF300-2R, avoiding disruption of other open reading frames in the region (pNG3, pNG1, J64R) (41). This recombinant virus is referred to as Benin-mNG. These virus genome structures were confirmed by PCR and sequence analysis across the sites of the insertion and deletion.

### Complete genome sequencing of the recombinant virus BeninΔA179L

The complete genome sequence of the first recombinant virus constructed, BeninΔA179L, confirmed the deletion of the A179L gene and the insertion of the β-glucuronidase reporter construct in its place as expected. We also detected two single nucleotide polymorphisms (SNP): a single nucleotide deletion in the noncoding region after A179L (position 48,741 on AM712239) and a single nucleotide deletion in the EP402R/CD2v gene at position 69,854 (AM712239) (Table 1). This was present at a high frequency in the sequence reads (97.8%), induced a predicted frameshift in the coding sequence of the EP402R/CD2v protein at residue 267, and introduced an in-frame termination codon in a different reading frame, 155 residues downstream. As predicted, this recombinant virus retained its hemadsorption activity since this is mediated by the extracellular domain of the protein. However, it is possible that the alteration in sequence of the cytoplasmic tail, including an extension, may affect functions dependent on that domain. Previous publications have indicated roles for the cytoplasmic domain in intracellular transport and inhibition of type I interferon induction, but it is unknown if mutation of this domain alone, or combined with other gene deletions, affects virus virulence.

**TABLE 1:** Changes in BeninΔA179L during whole genome sequencing^*a*^

	Reference position	Change type	Size (bp)	SNP^*b*^ %	Depth
Indel	48,152	Deletion of A179L from 48,152 to 48,534, replaced by GUS	383		
SNP	48,741	g.48741delT, SNP in the noncoding region between A179L and A137R; present in the transfer plasmid	1	98.7	1,000
SNP	68,364	c.798delT; p.S266fs; SNP in EP402R leading to frameshift	1	97.8	1,000

^*a*^ Reads were assembled under the variant analysis module (SeqMan NGen, DNASTAR) using the Benin 97/1 genome sequence obtained from NCBI (accession number: AM712239) as a reference guide.

^*b*^ SNP, single nucleotide polymorphisms.

In contrast, the A179L gene-deleted virus that expresses the mNeonGreen reporter (BeninΔA179L-mNG) was confirmed by Sanger sequencing of PCR fragments generated from the EP402R/CD2v gene region to have an identical EP402R/CD2v gene sequence to the parental Benin 97/1 isolate.

### Replication of BeninΔA179L deletion mutant viruses in cells

The replication kinetics of the BeninΔA179L virus in porcine bone marrow cells (PBMs) were compared with those of the parental Benin 97/1 virus over 5 d using a low multiplicity of infection (MOI) of 0.01. The virus was harvested from the combined supernatants and cell pellets and titrated. The results showed (Fig. 1C) that similar virus titers (10^6.3^ TCID_50_/mL) were obtained at day 1 post-infection. However, from day 2 post-infection, consistently lower titers were obtained from cells infected with BeninΔA179L. This difference was statistically significant at day 4 (*P* = 0.0004) with titers of 10^6.4^ TCID_50_/mL and 10^7.3^ TCID_50_/mL for BeninΔA179L and Benin 97/1, respectively. Thus, deletion of the A179L from Benin 97/1 impaired virus replication in PBMs over a multistep growth curve. A similar reduction in yield of the virus BeninΔA179L-mNG was observed over a multistep growth curve (Fig. 1E). This result confirmed that the frameshift mutation we observed in the cytoplasmic tail domain of the BeninΔA179L EP402R/CD2v gene was not the cause of the growth reduction. This was as expected since deletion of the EP402R/CD2v gene from virulent genotype II and genotype VIII genomes did not reduce virus replication in macrophages or virulence in pigs (42, 43). To determine if replication was also impaired over a single replication cycle, PBMs were infected at a high MOI (1.0) with BeninΔA179L or wild-type Benin 97/1, and virus was titrated from combined supernatants and cells at different times (1, 8, 16, 24, and 48 h post-infection). The results (Fig. 1D) showed no statistical difference between virus yields obtained at these time points. As expected, virus titers reached a plateau by 16 h post-infection, and these levels were maintained over the course of the experiment (approx. 10^6.8^ TCID_50_/mL). Thus, over a single cycle of replication, the total yield of the Benin∆A179L virus grown in PBMs was very similar to that of the parental virulent Benin 97/1 virus.

### Caspase 3/7 activation in macrophages infected with wild-type Benin 97/1 and BeninΔA179L viruses

**FIG 2:**
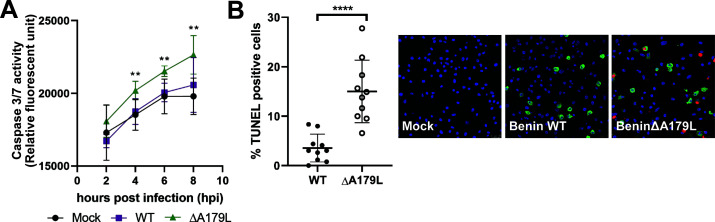
Increased pro-apoptotic signaling in cells infected with Benin∆A179L. (**A**) PBMs were infected with Benin 97/1 or BeninΔA179L at 0.5 MOI. The cells were loaded with 2 µM CellEvent Caspase-3/7 Green Detection Reagent, after which fluorescence measurements, indicative of caspase 3/7 activity, were taken 2, 4, 6, and 8 h post-infection (hpi). The y-axis shows relative fluorescence units, and the x-axis shows hours post-infection. Significance was determined by a two-way analysis of variance between Groups within each time point relative to the mock-infected cells. (**B**) PBMs were infected with Benin or BeninΔA179L at 0.25 MOI for 16 h. The cells were stained for TUNEL (red) and ASFV p30 (green). Cell nuclei are labeled with Hoechst 33342 (blue). Representative images are displayed. Scale bar = 25 µm. Percentage of TUNEL-positive cells calculated/field of view. Ten fields of view were counted for each virus. The significance was determined by a paired *t*-test. Data are expressed as mean ± SD.

The antiapoptotic activity of the exogenously expressed ASFV A179L protein has been established, but the impact of this protein during infection of cells is not known (21). Therefore, we examined the difference between the wild-type virus and the A179L deletion mutant on apoptotic signaling in infected PBMs, determined by caspase 3/7 activity. A179L is expressed at early times post-infection (38); therefore, we measured caspase 3/7 activity from 2 to 8 h post-infection. PBMs were mock-infected or infected with Benin 97/1 or BeninΔA179L viruses at an MOI of 0.25. The cells were loaded with 2 µM CellEvent Caspase-3/7 Green Detection Reagent for 1 h prior to infection, and fluorescence was measured in live cells every 2 until 8 h post-infection (Fig. 2A). There was no significant difference in caspase 3/7 activity in wild-type Benin 97/1-infected cells relative to the mock-infected PBMs. In contrast, caspase 3/7 activity was significantly increased in BeninΔA179L cells from 4 h post-infection relative to mock-infected cells (Fig. 2A). Early expression of A179L in cells infected with wild-type virus probably contributed to the reduction of caspase activity to levels similar to mock-infected cells. This contrasted with infections with BeninΔA179L in which increased caspase activation was observed between 4 and 8 hpi.

### Deletion of the A179L gene results in increased DNA fragmentation as measured by the TUNEL assay

To provide further evidence that greater levels of apoptosis are induced in cells infected with BeninΔA179L, we used an additional assay, DNA fragmentation by TUNEL staining. This assay detects apoptotic cells at a late stage. At late times, 16 h post-infection, the number of apoptotic cells was significantly greater in cells infected with BeninΔA179L than in cells infected with wild-type Benin or mock-infected cells (Fig. 2B).

### Deletion of the A179L gene results in increased cell surface binding of Annexin V

Exposure of plasma membrane phosphatidylserine during apoptosis allows the binding of Annexin V. To confirm that deletion of the A179L gene also results in increased activation of apoptosis, as measured by the binding of Annexin V to cell surfaces, we used live cell imaging over an extended time course with the IncuCyte S3. Purified PBMs were infected at a low MOI (0.1) with wild-type or A179L gene-deleted viruses, each expressing the mNeonGreen reporter under control of the ASFV early p30 promoter (BeninΔA179L-mNG or Benin-mNG). Binding of Annexin V red fluorescent reagent to live cell surfaces was visualized every 2 h for 48 h. In parallel, infected cells were monitored by the expression of mNeonGreen.

**FIG 3:**
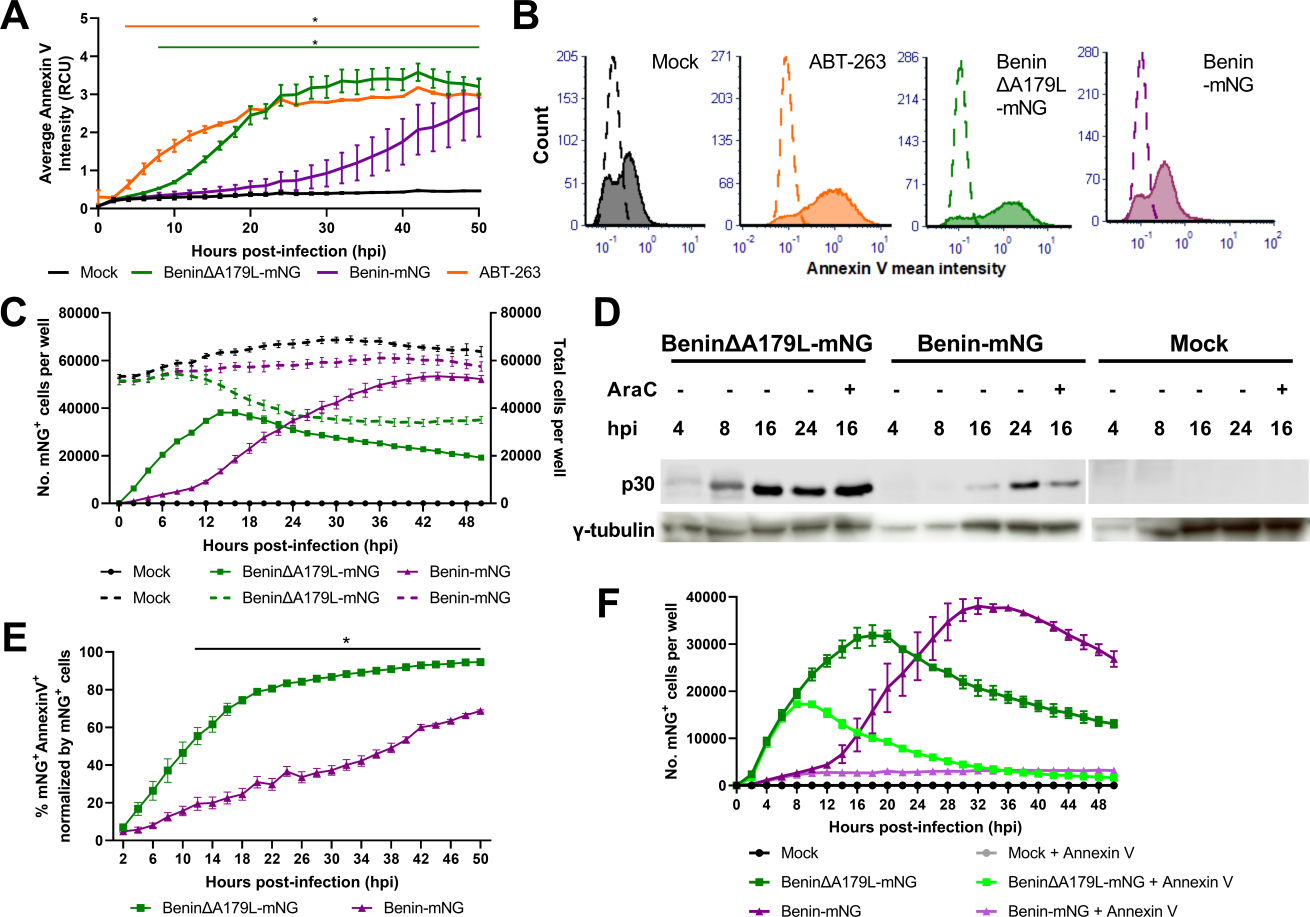
Cell surface Annexin V is induced at early times post-infection with gene-deleted BeninΔA179L-mNG compared to Benin-mNG. Purified PBMs were infected with Benin-mNG or BeninΔA179L-mNG at 0.1 MOI. Annexin V was added to cells immediately, and cells were monitored on the IncuCyte S3 live cell imaging system, imaged every 2 h for 48 h. (**A**) Using the adherent cell module, the average Annexin V intensity over time was measured. (B) The mean Annexin V intensity at 22 hpi for mock-infected, ABT-263-treated, and Benin-mNG- or BeninΔA179L-mNG-infected cells. **(C)** The number of cells expressing mNeonGreen (solid lines) and the total number of cells (dashed lines) in green for BeninΔA179L-mNG and purple for Benin-mNG-infected cultures. (**D**) Western blot analyses for ASFV early protein p30 in BeninΔA179L-mNG-, Benin-mNG-, and mock-infected PBMs at different time points after infection. Cytosine arabinoside (AraC)-treated cell lysates were also included. In parallel, a cellular loading control, γ-tubulin, was included. **(E)** The percentage of infected, apoptotic cells (mNeonGreen^+^Annexin^+^). A two-way analysis of variance was used to evaluate the differences between groups over time. **(F)** The number of infected cells (mNeonGreen^+^) after treatment of viruses with Annexin V.

Increased Annexin V binding to BeninΔA179L-mNG-infected cells was detected from early times, about 4 to 6 hpi, reaching a peak by 16 to 20 hpi and maintaining a plateau or gradually declining after that time point. In contrast, in the control Benin-mNG-infected cells, a lower amount of Annexin V binding was observed, increasing much more gradually after infection and continuing to rise during the 48-h culture period (Fig. 3A). As a positive control for the induction of apoptosis, ABT-263-treated cells were monitored in parallel. ABT-263 acts as a mimetic of BH3 domains and inhibits antiapoptotic members of the BCL-2 family, including BCL-2, BCL-x_L_, and BCL-W. In these treated wells, as expected, we also observed a substantial increase in Annexin V levels from four hpi (Fig. 3A).

Figure 3A shows the average Annexin V intensity per well. This may represent an increased number of Annexin V-positive cells in each well and/or increased Annexin fluorescent intensity in the positive cells. To better understand this finding, the Annexin V mean fluorescent intensity (MFI) per cell was examined at 22 hpi, when the ratio of Annexin V levels between BeninΔA179L-mNG- and Benin-mNG-infected wells was the greatest. Annexin V binding intensity in PBMs infected with Benin-mNG was comparable to that of the mock-infected cells, whereas a dramatic shift in MFI was observed in cells infected with BeninΔA179L (Fig. 3B). In fact, this shift was similar to the one observed in the apoptosis-inducer ABT-263-treated PBMs (Fig. 3B).

The number of BeninΔA179L-mNG-infected cells, assessed by expression of mNG, increased rapidly after infection and reached a peak by 16 hpi, thereafter declining gradually. In contrast, the numbers of Benin-mNG-infected cells increased gradually. By 16 hpi, the numbers of BeninΔA179L-mNG-infected cells were about double those of Benin-mNG. By 22 to 24 hpi, the numbers were similar in both infections, but the numbers of Benin-mNG-infected cells continued to rise and reached a plateau by about 36 hpi (Fig. 3C, solid lines). The results indicate an increase in BeninΔA179L-mNG virus uptake and early gene expression in cells (Fig. 3D). One possibility is that this may be mediated by the binding of phosphatidylserine, incorporated into the virus’s external envelope, to receptors on the host cell. The virus growth curves in Fig. 1C and E indicate similar levels of virus replication up to 1 d post-infection; thus, the increased numbers of mNeonGreen-expressing cells observed in BeninΔA179L-mNG-infected cultures post-infection do not apparently result in an increase in infectious virus progeny, perhaps due to abortive infection caused by earlier induction of apoptosis. We also compared total numbers of cells in cultures to determine if infection with BeninΔA179L-mNG caused a depletion in cell numbers (Fig. 3C, broken lines). This showed similar numbers of cells in cultures mock-infected or infected with either virus until about 12 hpi, and after that, the numbers of cells in mock-infected or Benin-mNG cultures remained relatively constant, whereas the numbers in the BeninΔA19L-mNG cultures decreased by about 25% at 24 hpi and then remained constant. The decrease in the number of cells infected with BeninΔA179L-mNG was closely correlated with the number of cells remaining in culture, indicating that cell death limited virus replication.

We also evaluated the percentage of mNeonGreen-expressing cells that bound to Annexin V to estimate the proportion of cells that were both infected and apoptotic. As shown in Fig. 3E, the percentage of infected and Annexin V-bound cells was also higher following infection with BeninΔA179L-mNG than with Benin-mNG. This was observed from four hpi, although the greatest difference in the percentage of apoptotic cells in cultures infected with these viruses was from about 12 hpi (Fig. 3E).

### Annexin V binding to virus preparations inhibits entry of BeninΔA179L-mNG and Benin-mNG

The increased infection rates observed in BeninΔA179L-mNG compared to Benin-mNG cells at early time points after infection were postulated to be due to viral apoptotic mimicry, a phenomenon seen in several viruses including orthopoxvirus, dengue virus, and Ebolavirus, by incorporating phosphatidylserine into their membranes, thus facilitating viral entry following binding to specific receptors at the cell membrane (44). Since we did not purify extracellular virions, it is also possible that apoptotic bodies loaded with ASFV virions may carry and deliver ASFV virions to uninfected cells. In a simple blocking experiment, we incubated BeninΔA179L-mNG and Benin-mNG viruses and mock clarified supernatant with Annexin V to block, if any, phosphatidylserine incorporated in the virions. These treated viruses were monitored alongside nontreated viruses in the same buffers/media composition on the IncuCyte. As seen previously, the number of BeninΔA179L-mNG-infected cells increased rapidly in both treated and nontreated samples as early as 4 hpi (Fig. 3F). Nonetheless, the numbers of infected cells in the treated BeninΔA179L-mNG started to decline much earlier at 10 hpi than those in the untreated BeninΔA179L-mNG-infected cells at 22 hpi. The impact of Annexin V pretreatment was even more dramatic in the case of Benin-mNG, as the number of infected cells stayed constant (~3,000) in the Annexin V-treated Benin-mNG samples up to 48 hpi compared to the untreated Benin-mNG, in which the number of infected cells increased from 12 hpi to reach a peak of ~37,704 infected cells at 36 hpi. The reasons why the Annexin V treatment was not so effective in reducing uptake of the BeninΔA179L-mNG virus at early times compared to Benin-mNG are not known. It is possible that the concentrations of phosphatidylserine in BeninΔA179L-mNG virus preparations are greater, so Annexin V binding is not saturating. We observed similar kinetics across a range of Annexin V concentrations between 2.5 and 10 μg/mL; it is possible that higher levels are required to achieve complete blocking. Further research is required to investigate this observation.

### Immunization of pigs with BeninΔA179L and challenge with virulent Benin 97/1—scoring of clinical signs and macroscopic lesions

One group of four pigs was immunized intramuscularly with 10^3^ TCID_50_/mL BeninΔA179L (Group D) and boosted with 10^4^ TCID_50_/mL of the virus on both 12 and 20 d post-immunization. These pigs (Group D) and a control group (Group F) of three nonvaccinated pigs were challenged with parental Benin 97/1 at 42 d post-immunization with 10^4^ TCID_50_/mL by the intramuscular route.

**FIG 4:**
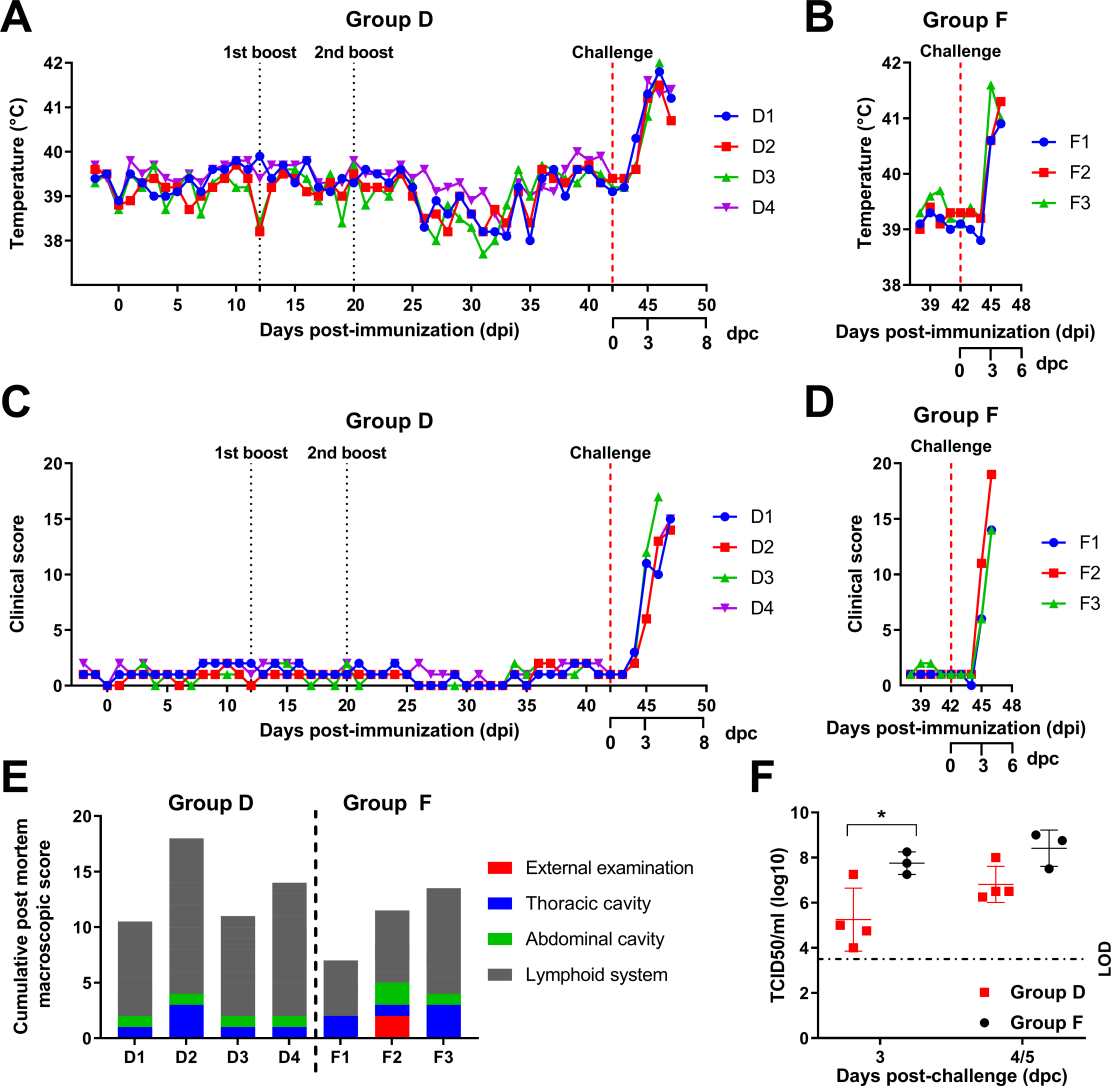
Temperatures, clinical scores, post-mortem macroscopic lesion scores, and viremia in pigs immunized with BeninΔA179L and challenged with virulent Benin 97/1. Rectal temperatures were recorded daily for Benin∆A179L-immunized pigs (Group D) (**A**) and nonimmunized naïve control pigs (Group F) (**B**). Cumulative clinical scores based on clinical signs observed daily are shown on panels C (Group D) and D (Group F). An additional axis shows the days post-challenge (dpc) for A-D. (**E**) Lesions observed were scored, and a cumulative score is shown for each pig. External examination (red bar) includes general body condition and conjunctiva. Lesions in the thoracic cavity (blue bar) include the presence of thoracic exudates as well as lesions affecting the cardiorespiratory system. Lesions in the abdominal cavity (green bar) include the presence of ascites along with lesions affecting the gastrointestinal system, including the stomach, intestines, liver, and gallbladder. Finally, lesions depicted by the gray bar include pathology observed in lymphoid tissues, such as the tonsils, thymus, spleen, and various lymph nodes. (**F**) Levels of infectious virus were measured for nonvaccinated pigs (Group F) and Benin∆A179L-immunized pigs (Group D) by titration of post-challenge whole blood collected in EDTA. No virus was detected before challenge in Group D pigs. A two-way analysis of variance with Šídák’s multiple comparisons test was performed to evaluate the differences between viremias in Group D and Group F. Significant differences are represented by an asterisk, where * is *P* < 0.05.

Rectal temperatures and clinical signs were recorded daily for all pigs. After the initial immunization and the first and second boosts with BeninΔA179L, the pigs showed similar average body temperatures with no increase in temperature or other clinical signs (Fig. 4A and C). At 3 d post-challenge, all pigs in Group D (BeninΔA179L) developed temperatures above 40.5°C and exhibited other signs typical of acute ASF, including lethargy and reduced eating (Fig. 4A and C). Clinical scores varied from 6 to 12, rising to 14–15. One pig was euthanized on day 4 post-challenge, and the remaining three pigs were euthanized at 5 d post-challenge at a moderately severe humane endpoint. The Group F (control) pigs displayed an increase in temperature (40.6°C–41.6°C) and other clinical signs typical of acute ASF by 3 d post-challenge, and all were euthanized at a moderately severe humane endpoint 4 d post-challenge (Fig. 4B and D).

All pigs in Group D (Benin∆A179L) and the control group (Group F) showed macroscopic lesions typical of acute ASFV at necropsy (Fig. 4E). The scoring of lesions (45) showed values between 10 and 17 in pigs in Group D and between 8 and 15 in Group F.

### Virus levels in blood

No infectious virus was detected in the blood of pigs in Group D before the challenge. At 3 d post-challenge, virus was detected in blood from the pigs in Group D and the control pigs in Group F. Pigs in Group F displayed a significantly higher titer of virus (mean: 10^7.8^ TCID_50_/mL) than those in Group D (mean: 10^5.3^ TCID_50_/mL) (*P* = 0.0149). There was no significant difference in levels of virus in the blood between the groups at the time of termination, 4 or 5 d post-challenge (Fig. 4F).

### Immune response in immunized pigs as measured by interferon gamma enzyme-linked immunospot and enzyme-linked immunosorbent assays

**FIG 5:**
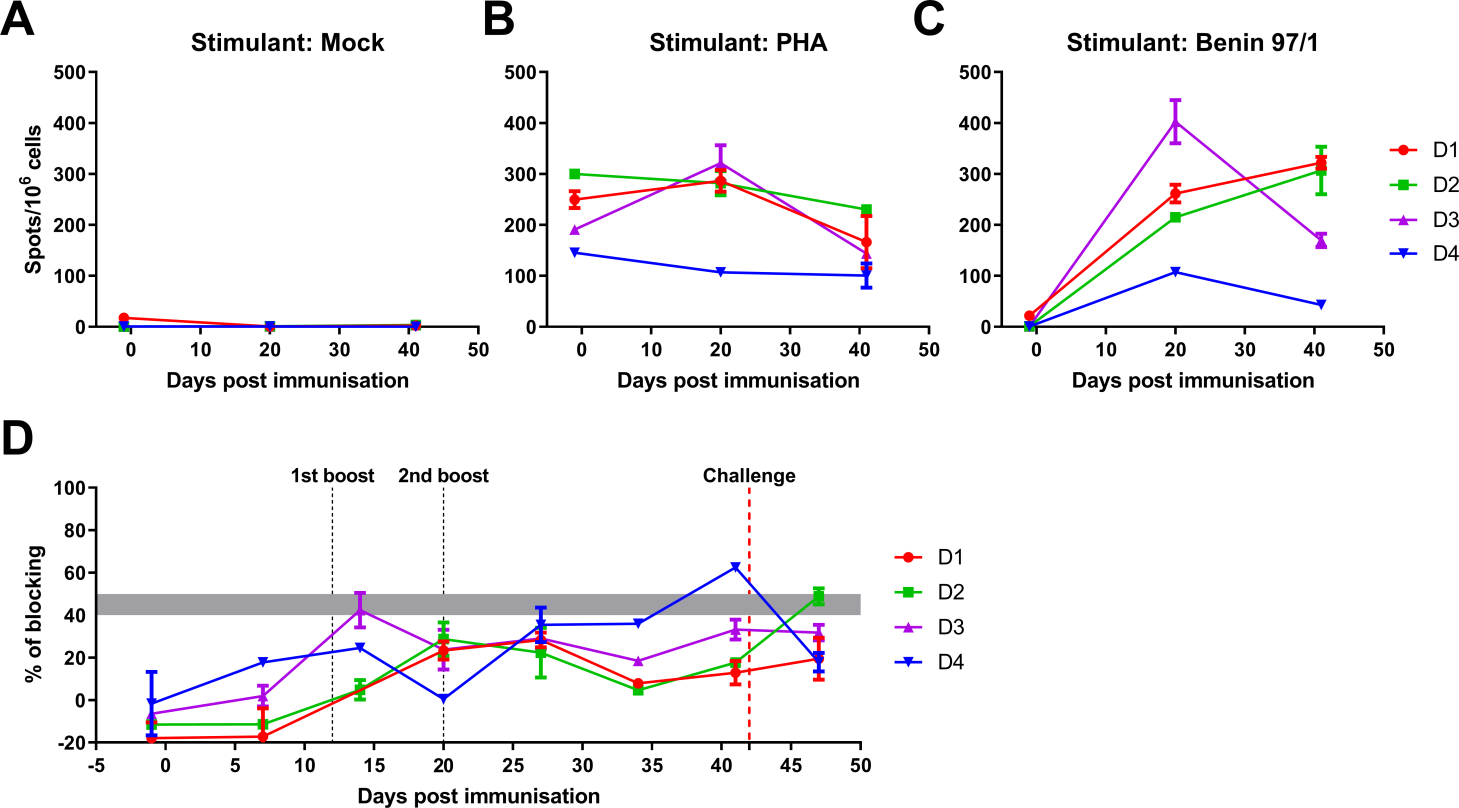
Immune responses of pigs immunized with Benin∆A179L and challenged with the virulent Benin 97/1. (**A-C**) The numbers of IFN-γ-producing cells in PBMCs of Group D pigs were measured at three time points: preimmune, prebooster, and prechallenge by ELISpot assays. PBMCs were stimulated with inoculum from mock-infected cells as a negative control (**A**), phytohemagglutinin (PHA) as a positive control (**B**), and a Benin 97/1 isolate (**C**). Results are presented as mean frequencies of IFN-γ-producing cells per million PBMC of individual pigs. (**D**) ASFV-specific antibody responses of Group D pigs were measured on different days after immunization and challenge using a blocking enzyme-linked immunosorbent assay against ASFV VP72. Results are presented as a percentage of blocking, where values above 50% blocking were considered positive antibody responses, while anything below 40% was considered negative. Samples with blocking between 40% and 50% were considered doubtful.

The response of peripheral blood mononuclear cells (PBMCs), isolated from pigs pre- and post-immunization and boosted with BeninΔA179L, to stimulation with ASFV Benin 97/1 was measured by interferon gamma (IFN-γ) enzyme-linked immunospot (ELISpot) assays. At the second boost, 20 d post-immunization, the numbers of IFN-γ-producing cells ranged between 100 and 400 IFN-γ-producing cells per million cells and decreased to approximately 40–300 million before challenge (Fig. 5C). Pigs D3 and D4 displayed a decrease in IFN-γ-producing cells prechallenge compared to the time of the second boost at 20 d post-immunization. Pig D3, which was terminated at 4 d post-challenge as opposed to other pigs in Group D, which terminated 5 d post-challenge, displayed the highest levels of IFN-γ-producing cells (approximately 400 IFN-γ-producing cells per 10^6^ cells) at 20 d post-immunization. This value decreased prior to the challenge (approximately 170 spots per 10^6^ cells). In contrast, the number of IFN-γ-producing cells from pigs D1 and D2 increased between the second boost and prechallenge (Fig. 5A).

A commercially available blocking enzyme-linked immunosorbent assay (ELISA) assay failed to detect antibody responses to ASFV p72 capsid protein above the threshold cut-off value throughout the experiment, with the exception of pig D4 just before challenge (Fig. 5B). In parallel experiments, pigs immunized with a DP148R gene -deleted Benin virus developed much higher humoral and cellular responses (46).

## DISCUSSION

Host cells respond to virus infection by activating a range of defenses to limit replication and induce both innate and adaptive immune responses. Signals induced by virus infection can activate apoptosis through several pathways. These include extrinsic signals, such as tumor necrosis factor-alpha, which binds to cell surface receptors to activate downstream apoptosis pathways. Intrinsic signals can activate pro-apoptotic BCL-2 family members, leading to loss of mitochondrial outer membrane integrity, release of cytochrome *c*, and initiation of caspase 3 and downstream caspase-mediated cleavage. Viruses have evolved a battery of mechanisms to inhibit apoptosis. ASFV codes for several apoptosis inhibitory proteins, including a member of the IAP family of proteins, A224L (22, 47), inhibitors of stress-induced apoptosis, including DP71L (23, 48), and the BCL-2 family member, A179L (21, 33, 49).

Our results confirmed that, as predicted for a BCL-2 antiapoptosis family member, caspase 3 and 7 activities increased at early times post-infection of macrophages with the recombinant virus from which the A179L gene was deleted, Benin∆A179L, compared to infections with the wild-type Benin 97/1 virus or in mock-infected macrophages. This result was expected since A179L is expressed at early times post-infection and is consistent with previous data showing that A179L acts as an antiapoptotic member of the BCL-2 family. Previous reports (50) and the Annexin V staining (Fig. 3A) showed that apoptosis was induced at later times following infection with wild-type virus containing an intact A179L gene. Delaying apoptosis induction until late can allow more time for virus replication, resulting in higher yields of infectious progeny. Downstream activation of effector caspases ultimately results in the induction of apoptosis. We confirmed by two additional assays that the downstream activation of apoptosis was increased in cells infected with the A179L gene deletion mutant compared to wild-type or mock-infected cells. In one assay, we confirmed, by binding of Annexin V, that cell surface phosphatidylserine, an early indicator of apoptosis, was increased following infection of purified PBMs with the A179L deletion virus BeninΔA179L-mNG compared to Benin-mNG. Secondly, we observed a significant increase in induction of apoptosis, as measured by DNA fragmentation using the TUNEL assay, post-infection of purified PBMs with Benin∆A179L compared to wild-type Benin 97/1. We concluded that deleting the A179L gene reduced antiapoptotic and pro-survival activity in infected cells and resulted in increased caspase 3 and 7 activities and downstream apoptosis. Binding of A179L to several pro-apoptotic members of the BCL-2 family has previously been described, and removal of this interaction is likely to be the mechanism involved in increasing apoptosis. In previous studies (50), induction of caspase 3 was increased in early times post-infection of porcine macrophages with the attenuated NHP68 isolate compared to the virulent Lisbon 60 isolate. Similar levels of apoptosis were observed following infection with both of these viruses at late times post-infection. In this previous study, the late induction of apoptosis was also observed, although at reduced levels, in the presence of the caspase 3 inhibitor zVAD-fnk. Thus, apoptosis observed at late times post-infection was probably mediated by other pathways in addition to those induced by caspase 3 activation (51).

We also showed that deleting the A179L gene from the genome of a virulent isolate, Benin 97/1, significantly reduced virus replication in macrophages over a multistep growth curve. However, over a single-cycle growth curve, the yield of infectious viruses was very similar to the wild-type Benin 97/1 virus. Surprisingly, live cell imaging, to detect infection of cells with recombinant viruses expressing an mNeonGreen fluorescent reporter, showed that the percentage of cells infected with the A179L gene-deleted virus BeninΔA179L-mNG, compared to Benin-mNG, was much higher from very early times, about 40% at 6 hpi, and reached a peak of about 80% by about 16 h post-infection. Thereafter, the cell numbers declined, but infection rates remained similarly high until about 30 hpi and then declined. In contrast, the numbers of cells infected with Benin-mNG, measured by the expression of mNG, were much lower. Less than 10% of cells were infected at 6 hpi, but the percentage increased steadily, reaching a plateau of ~80% mNG-expressing cells by about 40 h post-infection. Since the cells were infected with the same low MOI (0.1) of the BeninΔA179L-mNG and Benin-mNG viruses, we may have expected that similar numbers of cells would express the mNG fluorescent protein during the first cycle of replication. Although a higher number of cells were observed to be expressing mNG and therefore infected with BeninΔA179L-mNG, similar yields of infectious virus were obtained over a period up to 16 to 20 hpi, as shown in Fig. 1E. A likely explanation for this observation is that a higher proportion of the virions obtained from cultures infected with BeninΔA179L-mNG can enter cells efficiently and express proteins but do not produce fully infectious progeny, perhaps due to an earlier onset of apoptosis. Further investigation is required to confirm this hypothesis. We predict that if we had normalized infections to genome copy numbers rather than infectious virions, we would still have observed increased infection and early gene expression in cells infected with BeninΔA179L-mNG compared to Benin-mNG, although probably not to such an extent.

The higher number of mNeonGreen-positive cells at early times post-infection with the BeninΔA179L-mNG deletion mutant virus shows a higher entry rate of the virus and initiation of infection. ASFV acquires an external envelope derived from the host plasma membrane as it buds from host cells (1, 52). Virus preparations may potentially contain apoptotic bodies carrying infectious ASFV virions, also contributing to virus entry, although we have not investigated this. We observed increased Annexin V binding to cells infected with the BeninΔA179L-mNG deletion mutant from earlier times post-infection onward compared to those infected with the Benin-mNG virus, which has an intact A179L gene. Thus, increased incorporation of phosphatidylserine into the external ASFV envelope of these virions is predicted. Members of several enveloped virus families are known to incorporate phosphatidylserine into their membranes, resulting in enhanced cell entry by “apoptotic mimicry” mediated by binding to phosphatidylserine receptors on the host cell membrane (44, 53, 54). ASFV has been shown to enter cells both by receptor-mediated endocytosis and macropinocytosis. The latter is constitutive in macrophages and does not depend on virus-specific receptors (55, 56). Possibly apoptotic mimicry may explain increased infection rate of cells infected with the virus lacking A179L gene. Potential apoptotic mimicry could also extend the cellular tropism of ASFV. It may also shield virions from antibodies, contributing to the failure to detect fully neutralizing antibodies observed in other studies. Since mature ASFV virions lacking an extracellular membrane are also infectious, they would not be expected to have surface phosphatidylserine exposed on their surface and instead are likely to enter cells by receptor-mediated endocytosis. However, apoptotic bodies also have surface phosphatidylserine, so the spread of intracellular virions incorporated in apoptotic bodies and taken up by phosphatidylserine receptors on uninfected macrophages could provide a route for increased spread of these virions. We have not evaluated the proportion of virions in our preparation that contain an external envelope or are present in apoptotic bodies, so further evidence would be required to investigate these hypotheses.

The reduced virus spread we observed in PBMs infected with viruses lacking the A179L gene at a low MOI over a multistep growth process resulted at least in part from declining cell numbers. Since a high proportion of cells were infected, the decline in cell numbers was probably due to the early induction of apoptosis in the infected cells and decreased numbers of virions reaching maturity. It is also possible that a lower release of virus from infected cells contributed to reduced spread, as documented for HIV-1. At the virus budding step, HIV-1 can be trapped on the cell surface by one family of phosphatidylserine-binding TIM family receptors, and this can be counteracted by HIV Negative Factor (Nef) by inducing the internalization of these receptors (57).

Immunization of pigs showed that deletion of the A179L gene greatly attenuated the virulent Benin 97/1 virus. No clinical signs and no viremia were observed following immunization or boost of pigs with BeninΔA179L. Anti-p72 ASFV antibodies were not detected except in one pig, which had very low levels at one time point. Low cellular immune responses were induced, as measured by IFN-γ ELISpot assays, but the pigs were not protected against challenge with the parental virulent virus Benin 97/1. Significantly lower viremia was detected in pigs immunized with BeninΔA179L 3 d post-challenge compared to the control nonimmunized pigs, but on later days post-challenge, no difference in levels of viremia was observed between immunized and control pigs. We assume that *in vivo*, insufficient virus replication occurred to induce an effective protective immune response.

In contrast to our results with A179L, deletion of other apoptosis inhibitors from the ASFV genome does not reduce virus replication in cells or virulence in pigs. ASFV IAP family member, A224L, is proposed to interact directly with the processed fragment of caspase 3 (22) to inhibit apoptosis downstream of caspase 3. Despite these antiapoptotic activities, deletion of the A224L gene from a virulent isolate did not reduce virus replication in porcine macrophage cultures or the induction and magnitude of apoptosis. Deletion of this gene also did not reduce virulence in infected pigs (58).

The ASFV DP71L protein inhibits apoptosis pathways mediated by the transcription factors CHOP/ATF4 (23, 48). The impact of deleting the DP71L gene on virus virulence in pigs varies depending on the virus isolate. Thus, deletion from the E70 isolate reduced virulence in pigs, whereas deletion from the Malawi genotype VIII isolate did not reduce virulence (59).

Our results show that increased caspase 3 activity and induction of apoptosis in macrophages infected with the Benin∆A179L virus correlate with reduced replication and virus attenuation in pigs. However, we cannot exclude the role of other functions of the A179L protein, for example, inhibition of the autophagy regulator Beclin-1. ASFV does not encode other known BCL-2 family members, and thus A179L may be the only inhibitor of the mitochondrial pathway of apoptosis. This may explain the greater impact on virus replication and virulence of deleting the A179L gene compared to other apoptosis inhibitors. Our results demonstrate for the first time the important role played by the A179L BCL-2 family apoptosis inhibitor during virus replication in macrophages and in pigs. The results suggest that manipulation of apoptosis could provide a method to control virus replication by providing targets for antiviral drugs or helping in the design of novel vaccines.

## MATERIALS AND METHODS

### Viruses and virus titration

The Benin 97/1 genotype I (genome reference AM712239) virulent field isolate has been previously described (60). The Benin 97/1 isolate was cultured in PBMs in Earle's balanced salt solution (EBSS) (Sigma), supplemented with 4 mM Hepes, 10% heat-inactivated porcine serum (BioSera, France), 100 IU/mL penicillin, and 100 µg/mL streptomycin (61). Titration of the viruses was carried out by hemadsorption assay (presented as HAD_50_/mL) or by immunofluorescence using antibodies against ASFV early protein p30/CP204L (presented as TCID_50_/mL), calculated using the Spearman and Karber formula.

### Deletion of the A179L gene from the ASFV isolate Benin 97/1

A transfer vector was constructed containing fragments flanking the A179L gene from Benin 97/1 upstream and downstream. A reporter gene β-glucuronidase under control of the ASFV p72 promoter was inserted between these flanking regions. The left flanking region was amplified with the primers 5′-GCGCAAGCTTCAGAGGGCAAAGATGGCTCAACCAC-3′ and 5′-GCGCGGATCCGATTTCCCACGGCGGTCAAGAGGAG-3′. The obtained DNA fragment of 534 bp was inserted between the *Hin*dIII and *Bam*HI restriction sites within pcDNA3_VP72-GUS (positions 47,618 to 48,150 within the Benin 97/1 genome). The right flanking region includes 178 bp from A137R and was cloned using the primers 5′-GCGCGAATTCAGCGGCACCCTATATTTTTTTATTTAGG-3′ and 5′-GCGCCTCGAGCGGGGGTAAATAAAAGCTCC-3′. The right flanking region of 421 bp (positions 48,535 to 48,955 of the Benin 97/1 genome) was inserted between the *Eco*RI and *Xho*I restriction sites in pcDNA3_VP72-GUS. PAMs were infected with Benin 97/1. Infected cells were transfected with the transfer plasmid using TransIT-LT1 (Mirus Bio, Madison, WI, USA). Recombinant viruses Benin∆A179L, with a deletion between positions 48,151 and 48,535, expressing the β-GUS gene, were identified by incubation with 5-bromo-4-chloro-1H-indol-3-yl β-D-glucopyranosiduronic acid and purified by limiting dilution. The same flanking regions were used to produce BeninΔA179L-mNG, employing the mNeonGreen reporter to isolate recombinant viruses by single cell sorting as previously described (40). The control virus, Benin-mNG, was obtained using the same method, with the mNeonGreen reporter inserted in a noncoding area of Benin 97/1 between MGF300-1L and MGF300-2R.

### Complete genome sequencing of the recombinant virus BeninΔA179L

The virus was grown in a 175-mL flask of PBMs. Virus DNA was purified from lysed virions in cell supernatants after treatment with DNAse I to degrade cellular DNA using a modification of the procedure used previously (62). DNA was extracted from the supernatant using the MagAttract HMW DNA kit (Qiagen). DNA libraries were prepared using the Illumina DNA Prep Kit (Illumina) and sequenced on the MiSeq instrument using a 600-cycle v3 cartridge. A total of 6,085,518 reads were obtained, and 77.8% were assembled under the variant analysis workflow in SeqMan NGen (DNASTAR) when compared to the ASFV Benin 97/1 isolate (accession number: AM712239) with a median coverage of 3,490.16. The comparative analysis confirmed the deletion of the A179L gene from 1 to 383 bp (positions 48,152 to 48,534 of the Benin 97/1 genome) and the insertion of β-GUS (1,812 bp) under the control of the ASFV VP72 promoter (5′-atttaataaaaacaataaattatttttataacattatat-3′).

### Virus replication kinetics in cells

The Benin 97/1 and Benin∆A179L viruses were titrated in PBMs from two different pigs. The cells were infected at an MOI of 0.01 (multistep growth curve) or an MOI of 1 (single-cycle growth curve). The cells were incubated at 37°C for 1 h, and then the inoculum was removed. After gently washing with phosphate-buffered saline (PBS), fresh medium was added to the wells, and the cells were further incubated at 37°C up to 48 h post-infection for the single-cycle growth curve or for 1 to 5 d for the multistep growth curve. Cells and supernatants were collected at different times post-infection and subjected to three freeze-thaw cycles. Virus titers were determined as described above.

### Caspase 3/7 activity assay

Purified PBMs, seeded at 5 × 10^6^ cells/mL in black polystyrene Nunc 96-well plates (Thermo Fisher), were cultured in Roswell Park Memorial Institute (RPMI) medium supplemented with 10% heat-inactivated fetal calf serum (FCS), 100 IU/mL penicillin, 100 µg/mL streptomycin, and 100 ng/mL porcine colony stimulating factor -1 (CSF1) (Roslin Technologies) for 3 d. Prior to infection with the indicated virus at 0.25 MOI, the cells were loaded with 2 µM CellEvent Caspase-3/7 Green Detection Reagent (Thermo Fisher Scientific), according to the manufacturer’s instructions for kinetic assays and fluorescence in the live cells, indicative of caspase activity, recorded using a Synergy2 Multi-Detection Microplate Reader (BioTek) at the indicated time points post-infection.

### TUNEL assay to measure apoptosis

Purified PBMs, seeded at 3 × 10^6^ cells/mL in chambered slides (Ibidi, Germany), were cultured in RPMI supplemented with 10% heat-inactivated FCS, 100 IU/mL penicillin, 100 µg/mL streptomycin, and 100 ng/mL porcine CSF for 3 d. The cells were infected with Benin 97/1 or BeninΔA179L at 0.25 MOI for 16 h. The cells were fixed in 4% paraformaldehyde and washed with PBS, followed by terminal deoxynucleotidyl transferase-dUTP nick end labeling (Click-iT Plus TUNEL Assay for *In Situ* Apoptosis Detection, Thermo Fisher Scientific), according to the manufacturer’s protocol. The cells were subsequently stained with mouse anti-p30 C18 (63) for 1 h at room temperature (RT) and the corresponding goat antimouse IgG (H + L) cross-adsorbed secondary antibody, Alexa Fluor 488 (Thermo Fisher Scientific), at 1 μg/mL for 1 h at RT. Nuclei were labeled with Hoechst 33342, diluted in PBS at a final concentration of 5 µg/mL, and incubated at RT for 15 min. Imaging was performed using a Leica TCS SP8 confocal microscope with a 40× oil immersion objective. The total number of cells and TUNEL-positive cells was counted using ImageJ 1.x (64).

### Annexin V apoptosis assay

Purified PBMs were seeded at 1.25 × 10^5^ cells per well in 96-well plates. After 2 d, the cells were treated with IncuCyte Annexin V Red reagent at 1:100 dilution in EBSS and then immediately mock-infected or infected with either Benin-mNG or BeninΔA179L-mNG. Purified PBMs were also treated with 20 µM of ABT-263 (ChemCruz, Santa Cruz Biotechnology), a mimetic of BH3 domains, to inhibit members of the BCL-2 family, specifically BCL-2, BCL-x_L_, and BCL-W. Cells were monitored for 48 h using an IncuCyte S3 live-cell imaging system (Sartorius) in a 37°C incubator. Four image fields per well were captured every 2 h, and spectral unmixing was carried out as recommended, where 8% of the red fluorescence was removed from the green channel. Using the IncuCyte integrated software, the average Annexin V mean intensity was obtained over time, and repeated measures two-way analysis of variance (ANOVA) with Dunnett’s multiple comparison tests was used to compare the differences between the groups. To plot the MFI of Annexin V, raw cell-by-cell object data for the 22 hpi time point, where the ratio of Annexin V was highest between Benin and BeninΔA179L, were exported and analyzed using FCS Express 7. The percentages of mNeonGreen and/or Annexin-positive cells were calculated using the IncuCyte integrated software module based on a user-defined cell-by-cell classification. In a similar manner to analysis by flow cytometry, wells containing controls [(i) negative for mNeonGreen and Annexin V, (ii) single color controls—mNeonGreen positive only or Annexin V positive only, (iii) double positive for mNeongreen and Annexin V] were used to set up quadrants. Cells were then classified as (i) uninfected, nonapoptotic cells (mNG^−^AnnexinV^−^); (ii) uninfected, apoptotic cells (mNG^−^AnnexinV^+^); (iii) infected, nonapoptotic cells (mNG^+^AnnexinV^−^); and (iv) infected, apoptotic cells (mNG^+^AnnexinV^+^). Repeated measures two-way ANOVA with Šídák’s multiple comparison test was done to evaluate differences in the number of infected, apoptotic cells over the total infected cells between the groups. This experiment was repeated with purified PBMs from three different pigs.

### Annexin V blocking assay

Purified PBMs were seeded at 1.0 × 10^5^ cells per well in 96-well plates. After 2 d, BeninΔA179L-mNG, Benin-mNG, and mock-infected supernatants were diluted to achieve a 0.1 MOI in 1× Annexin Binding Buffer (BD Pharmingen). The viruses were then incubated with purified recombinant Annexin V (BD Pharmingen) at varying concentrations, 2.5–10.0 µg/mL, at 37°C for 1.5 h. These viruses were then added to the PBMs containing fresh complete medium, and the plates were monitored for 48 h using an IncuCyte S3 live-cell imaging system (Sartorius) in a 37°C incubator as described above.

### Western blotting

Purified PBMs were infected with BeninΔA179L-mNG and Benin-mNG at an MOI of 0.1. Cells were collected into tubes at 4, 8, 16, and 24 hpi and centrifuged at 400 × *g* for 10 min at 4°C. Cells were lysed in 4× Laemmli Sample Buffer (Bio-Rad) supplemented with 10% β-mercaptoethanol. Cell lysates were heated at 100°C for 5 min before running on a 12% mini-Protean TGX gel (Bio-Rad). After electrophoresis, protein was transferred to Amersham Hybond P 0.45 polyvinylidene difluoride (PVDF) blotting membranes (Cytiva). Blots were blocked with 5% blotto (5% milk in 0.1% Tween in 100% Tris-buffered saline (TBS)) and probed for ASFV p30 mouse monoclonal antibody and a cellular loading control, mouse monoclonal antiγ-tubulin (Sigma-Aldrich). After probing with either a secondary HRP-conjugated goat antimouse IgG antibody (CST) or the IRDye 800CW goat antimouse secondary antibody (LI-COR), target antibodies were detected via ECL Select (Amersham) or imaged on the Odyssey CLx Infrared imaging system (LI-COR), respectively.

### Immunization and challenge of pigs

Immunization and challenge of pigs with ASFV were carried out at The Pirbright Institute, Surrey, UK, in high-containment large animal facilities licensed at UK Specified Animal Pathogens Order Level 4. Animal experiments were ethically reviewed and carried out under the Animals Scientific Procedures Act, 1986, licensed by UK Home Office Project License PPL70/8852. Large White Landrace pigs of 15 to 20 kg weight were randomly assigned to Group D, which comprised four pigs that were immunized and boosted by the intramuscular route with the gene-deleted virus Benin∆A179L, then challenged by the intramuscular route with the virulent parental virus Benin 97/1 in parallel with a group of three control pigs (Group F). The schedule and doses of virus used are described in Results. Rectal temperatures and other clinical signs were recorded daily from before the day of immunization until termination. EDTA blood and serum samples were collected at different days post-immunization or challenge, as indicated in Results, to measure levels of virus, antibody, and cellular immune responses. Animals were euthanized at a moderate-severity endpoint. Macroscopic lesions were scored post-mortem as described previously (45).

### ASFV-specific antibody responses

A commercial blocking ELISA (Ingenasa PPA3 Compac) was used to measure the level of antibody responses against ASFV-p72 in serum, as per the manufacturer’s instructions. The following formula was used to calculate the percentage of blocking (PB): [(negative-control OD − sample OD) / (negative-control OD − positive-control OD)] × 100, where OD is the optical density. Samples were considered positive if the PB was above the cut-off value of 50% blocking.

### IFN-γ ELISpot assays

PBMCs were purified from heparinized blood using gradient centrifugation. The IFN-γ ELISpot assay has been previously described (65, 66). ELISpot plates (MAIPS4510; Millipore) were coated overnight at 4°C with 4 µg/mL antiporcine IFN-γ mAb P2F6 in 0.05 M carbonate-bicarbonate coating buffer and then washed with PBS. Cells were plated in duplicate at two different dilutions, typically 8 × 10^5^ and 4 × 10^5^ per well, in RPMI supplemented with 10% FCS, 1 mM sodium pyruvate, 50 µM 2-mercaptoethanol, 100 IU/mL penicillin, and 100 µg/mL streptomycin. Cells were then incubated overnight in a final volume of 200 µL with 10^5^ hemadsorption units of Benin 97/1 or an equivalent volume of mock inoculum (Fig. 5A), or 2.5 µg/mL phytohemagglutinin as a positive control (Fig. 5B). Cells were lysed by incubating for 5 min in water and then washing with PBS. Biotinylated antiporcine IFN-γ mAb (P2C11), followed by streptavidin conjugated to alkaline phosphatase, was used to visualize spots that were then counted using an ELISpot Reader System (AID). The number of spots per well was converted into the number of spots per million cells, and the mean of duplicate wells was plotted.

### Statistical analysis

Statistical analysis, as indicated, was performed using GraphPad Prism 8 software. A two-way ANOVA followed by Šídák’s or Dunnett’s multiple comparison test was used to evaluate differences between groups.
